# A portable microfluidic platform for rapid molecular diagnostic testing of patients with myeloproliferative neoplasms

**DOI:** 10.1038/s41598-017-08674-8

**Published:** 2017-08-17

**Authors:** Hua Wang, Xinju zhang, Xiao Xu, Qunfeng Zhang, Hengliang Wang, Dong Li, Zhihua Kang, Zhiyuan Wu, Yigui Tang, Zhenhua An, Ming Guan

**Affiliations:** 1 Deptartment of Laboratory Medicine, Huashan Hospital, Shanghai Medical College, Fudan University, Shanghai, 200040 China; 2 Deptartment of Physics, Fudan University, Shanghai, 200040 China; 3 Deptartment of Clinical Laboratory, Shanghai Tongji Hospital, Tongji University School of Medicine, Shanghai, 200065 China

## Abstract

The ability to simultaneously detect JAK2 V617F and MPL W515K/L mutations would substantially improve the early diagnosis of myeloproliferative neoplasms (MPNs) and decrease the risk of arterial thrombosis. The goal of this study is to achieve a point of care testing platform for simultaneous analysis of major genetic alterations in MPN. Here, we report a microfluidic platform including a glass capillary containing polypropylene matrix that extracts genomic DNA from a drop of whole blood, a microchip for simultaneous multi-gene mutation screening, and a handheld battery-powered heating device. The µmLchip system was successfully used for point-of-care identification of the JAK2 V617F and MPL W515K/L mutations. The µmLchip assays were then validated by mutation analysis with samples from 100 MPN patients who had previously been analyzed via unlabeled probe melting curve analysis or real-time PCR. The results from the µmLchip were in perfect agreement with those from the other methods, except for one discrepant result that was negative in the unlabeled probe melting curve analysis but positive in the µmLchip. After T-A cloning, sequences of cloned PCR products revealed JAK2 V617F mutation in the sample. The portable microfluidic platform may be very attractive in developing point-of-care diagnostics for MPL W515K/L and JAK2 V617F mutations.

## Introduction

Classical myeloproliferative neoplasms (MPNs), which include polycythemia vera (PV), essential thrombocythemia (ET), and primary myelofibrosis (PMF), are a subclass of hematological malignancies that feature clonally proliferateing blood cells. These diseases may evolve into severe anemia, leukemic transformation and other pathologies. Thrombosis is the most common complication and the major cause of death. A thrombotic event may be the presenting clinical feature that leads to the diagnosis of an MPN, but thrombosis can also occur before diagnosis or during the course of the disease. Several studies have described an increased risk of thrombosis before and after MPN diagnosis^1, 2^. A large proportion of MPN patients could potentially be diagnosed considerably earlier if proper investigations are performed when the abnormal blood values were observed^3^. Molecular testing plays a crucial role in each of these disease entities. JAK2 V617F and MPL W515K/L have been identified as the standard genetic markers for MPN diagnosis^4^.

The JAK2 V617F mutation is present in almost all patients with PV and in approximately 50% of the patients with ET and PMF. Moreover, the burden of mutant alleles influences the disease phenotype and is associated with a greater risk of thrombosis in both ET and PV, as well as with disease progression in PMF^5–7^. The role of the JAK2 V617F mutation, as a risk factor for vascular complications, has been evaluated in many studies, which have found variable results^8–10^. Furthermore, the demographic characteristic of ET and PMF patients may be useful for improving risk prediction and informing clinical screening and treatment strategies^11^. MPLW515 K/L mutations have been reported in approximately 5% of JAK2 V617F-negative PMF cases and approximately 1% of all cases of ET^12, 13^. Therefore, the ability to simultaneously detect the JAK2V617F and MPL W515K/L mutations would substantially improve the early diagnosis of MPN and decrease the risk of arterial thrombosis.

At present, the optimal approach for the simultaneous detection of low-abundance JAK2 V617F and MPL W515K/L mutations has not been defined. Innovative diagnostic assays that have high analytical sensitivity and are suitable for routine use are required. We have previously developed an ibLchip that performs genomic DNA extraction from whole human blood and loop-mediated isothermal amplification (LAMP), to permit visual detection of gene mutations^14^. However, this chip extracts only a very small amount of DNA and identifies only the JAK2 V617F mutation. Moreover, an electric hot plate is needed at several steps of the process, thus further limiting the application of this methodology at the point of care applications. Hence, development of a microfluidic platform for simultaneous point-of-care JAK2 V617F and MPL W515K/L mutations testing has become the urgent matter.

**Figure 1:**
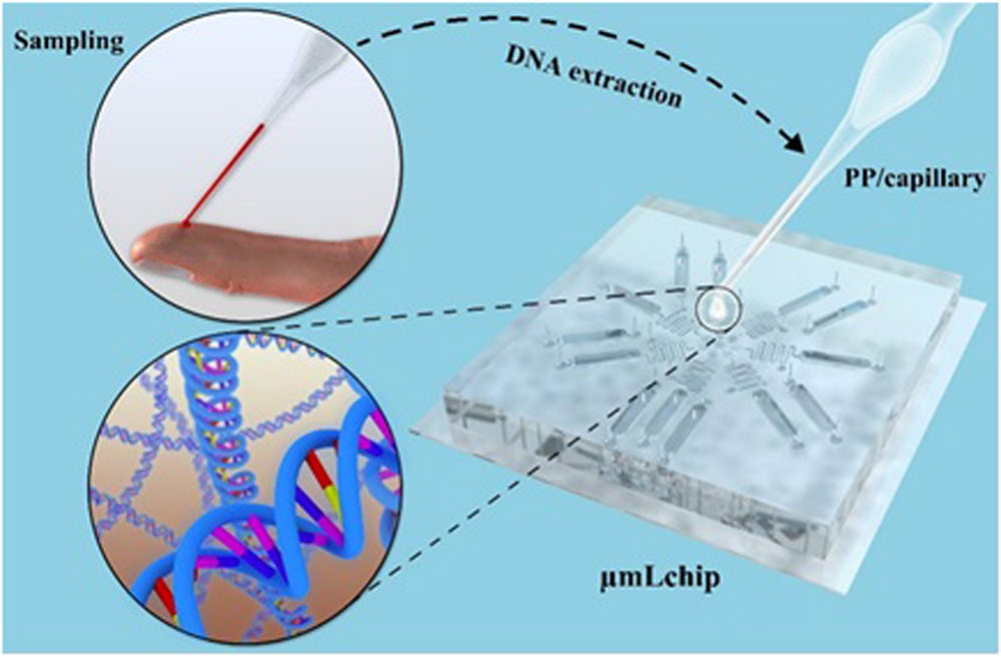
3D schematics of the PP/capillary and the µmLchip. The PP/capillary allows for peripheral blood collection and rapid DNA release for rapid and efficient extraction of genomic DNA from human blood. The µmLchip was successfully used for point-of-care identification of JAK2 V617F and MPL W515K/L mutations.

Given this need, our aim in this study was to develop a portable microfluidic platform for rapid molecular diagnostic testing of patients with myeloproliferative neoplasms. The platform includes a glass capillary containing polypropylene matrix for genomic DNA extraction from a drop of whole blood, a microchip for simultaneous multi-gene mutation screening, and a handheld battery-powered heating device. At present, on-chip DNA extraction is still a major challenge. Several groups have attempted to extract DNA from whole blood on a microchip^15–19^. However, those methods are still not commonly used at the point of care because they require specialized equipment (a syringe pump). The majority of on-chip extraction systems use a bind-wash-elute protocol. In a fully integrated microsystem, this elution step often causes the dilution of the extracted DNA, thus decreasing the sensitivity of the genetic analysis^20^. It is known that as the first step in the overall genetic analytical process, the DNA extraction determines the quality of the DNA template that can be provided to the following analytical steps. To overcome these challenges, we used a glass capillary of containing polypropylene matrix (PP) for DNA extraction. This capillary could perform peripheral blood collection and rapid DNA release. In addition, on the basis of based on our previous work regarding the combination of LAMP and microfluidics, we developed a LAMP microchip for the identification of JAK2 V617F and MPL W515K/L mutation in the peripheral blood of ET and PMF patients. We called it a µmLchip because it is namely a LAMP microchip for simultaneous multi-gene mutation screening. As shown in Fig. 1, the PP/capillary allows for peripheral blood collection and rapid DNA release for rapid and efficient extraction of genomic DNA from human blood. The µmLchip was successfully used for point-of-care identification of JAK2 V617F and MPL W515K/L mutation. Furthermore, because constant temperature is normally required for nucleic acid extraction and amplification, we developed a handheld battery-powered heating device to allow the use of LAMP in remote settings. Simple, rapid sample preparation techniques that do not require any additional user interaction are ideal for use of portable microfluidic platforms doctor’s offices, in the field, or at the bed-side.

It should be noted the landmark discovery of CALR Exon 9 mutations in 60–80% of JAK2 V617F- and MPL Exon 10-negative ET and PMF cases compels the inclusion of CALR mutation analysis into the molecular diagnostic algorithm for these MPNs^21^. However, CALR mutations were not included in our study, which may limit its clinical application. Further studies on analysis of detecting these mutations (JAK2 V617F, MPL W515K/L, CALR) on a microchip are going on in our group.

## Results and Discussion

### On-PP/capillary peripheral blood genomic DNA extraction

**Figure 2:**
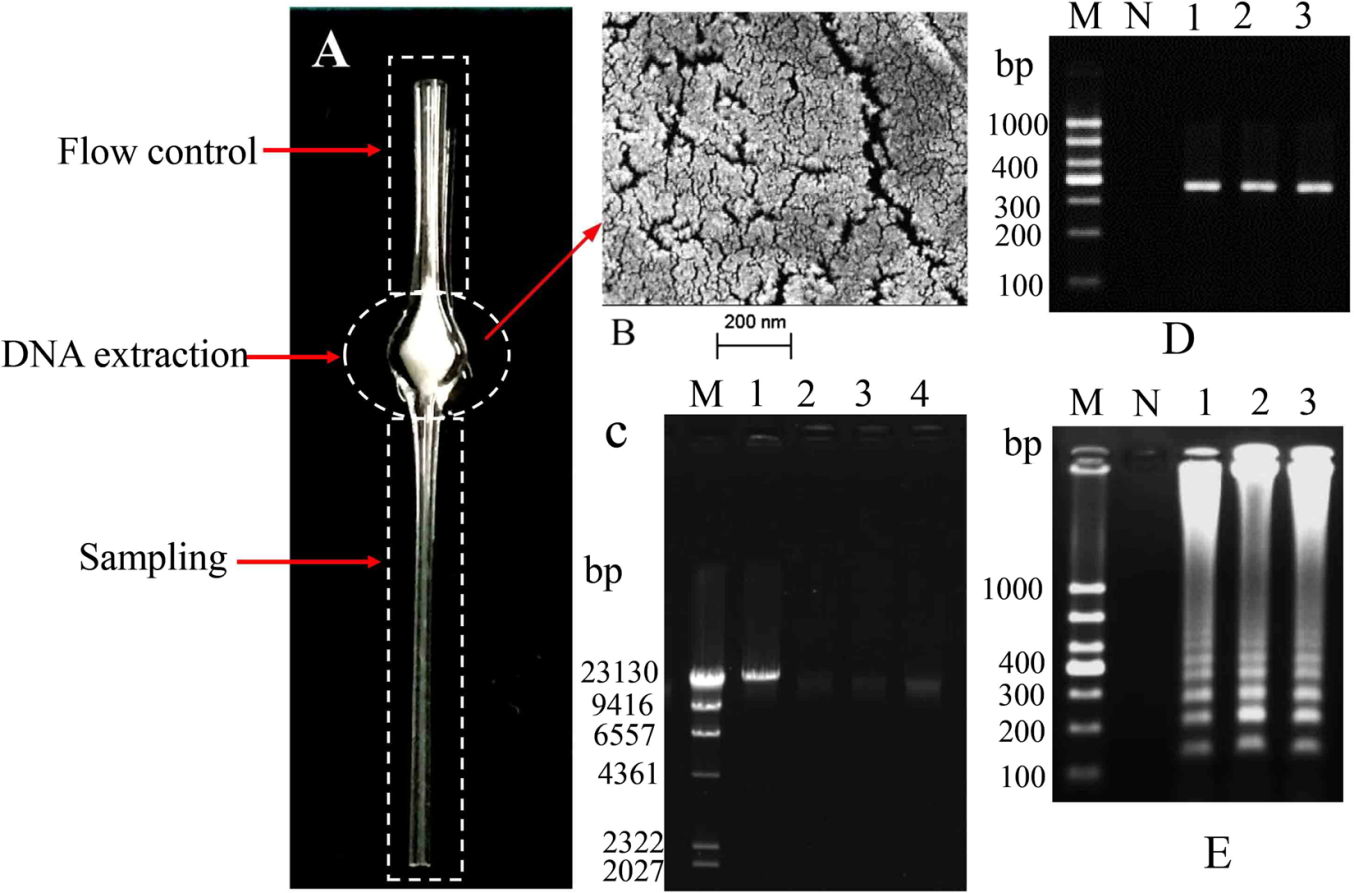
(**A**) Photograph of the PP/capillary. The glass capillary described here has three distinct functional domains, one for sampling, another for DNA extraction (the region filled with 8 mg of PP) and the last for controlling the flow of fluids. (**B**) Scanning electron micrograph of PP. (**C**) The results of electrophoresis in an agarose gel. Lanes 2–4: extraction of the genomic DNA from 5, 10, and 20 µL of whole bloeod, respectively. Lane M: DNA marker, λDNA/Hindβ; Lane 1: λDNA (50.0 kb). (**D**) Electrophoresis of PCR products amplified from 5, 10, and 20 µL of human peripheral blood extracted on the PP/capillary. Lane M: 1000 bp DNA marker; NC: negative control; Lanes 1–3: PCR-amplified results from 5, 10, and 20 µL of whole blood, respectively. (**E**) Electrophoresis of LAMP products amplified from 5, 10, and 20 µL of human peripheral blood extracted with the PP/capillary. Lane M: 1000 bp DNA marker; NC: negative control; Lanes 1–3: LAMP-amplified results from 5, 10, and 20 µL of peripheral blood, respectively.

To evaluate the ability of the PP/capillary to extract peripheral blood genomic DNA, the efficiency of the on-capillary DNA extraction by the device shown in Fig. 2 was tested using 5, 10 and 20 µL of human peripheral blood samples, according to the protocol described above. The DNA was then quantified with a Qubit® 2.0 Fluorometer. The results showed that on-PP/capillary DNA extractions successfully produced 40 ± 2.5, 78 ± 4.2 and 155 ± 5.8 ng (n = 3) of DNA from 5, 10 and 20 µL of blood, respectively. To verify the DNA extraction efficiency, 2 µL of DNA extracted from 5, 10, or 20 µL of whole blood samples was amplified by PCR. The human control β-globin primers used in DNA template amplification were 5′-AGTCAGGGCAGAGCCATCTA-3′and 5′-TTAGGGTTGCCCATAACAGC-3′. The size of the fragment generated was 385 bp. PCR analysis confirmed that the DNA extraction process was successful as shown in Fig. 2D. To test whether amplifiable DNA suitable for subsequent genetic analysis could be extracted from the whole blood sample, 2 µL of the DNA extracted from 5, 10, or 20 µL of whole blood samples was amplified by using the LAMP reaction (MPL W515 wild type primers). Ladder-like bands on the agarose gel, which are characteristic of LAMP, were observed after target amplification (Fig. 2E, lines 1–3), thus indicating successful DNA extraction and amplification. To further examine the integrity of the DNA obtained by the PP/capillary extraction, agarose gel electrophoresis of the genomic DNA from whole blood were performed. The results of the agarose gel electrophoresis are shown in Fig. 2C. Lanes M, 1, 2, 3, and 4 correspond to λDNA/HindIII, λDNA, 5 µL of blood genomic DNA, 10 µL of blood genomic DNA, and 20 µL of blood genomic DNA, respectively. As shown in lanes 2–4, bands of approximately 30.0 kb were observed. The results suggested that the high integrity of the genomic DNA from the PP/capillary extraction. To provide a more comprehensive validation of the reliability of the DNA extraction system, the more replicates were tested from 10 patients with anemia and 10 healthy controls peripheral blood, at these volumes. Each sample was tested three times. The DNA concentrations of patients with anemia were significantly lower than that of healthy control. There were apparent significant differences between the two groups (Fig. SI4). Talking into account the fact that the DNA concentration of some special populations would be expected to be lower, 10 µL peripheral blood was chosen for subsequent clinical sample analysis.

To understand the principle of the method, scanning electron microscopy (SEM) was performed using a Zeiss Supra 55 VP field emission SEM (Oberkochen, Germany) to visualize the surface morphologies of the PP. The PP was coated with gold (Au3501, 99.9999%, Beijing Chino Far East Technology Co., Ltd) by using thin-film devices (NANO36, Kurt J. Lesker Company^®^) before imaging, to prevent charging of the surface. As shown in Fig. 2B, the surface of the PP had a porous structure that created a large surface area for DNA extraction. The mechanism of DNA extraction from blood by the PP/capillary is believed to be as follows: the cells in blood are lysed. The exposed genomic DNA molecules are trapped in the porous structure of the PP, whereas the cell debris, hemoglobin, and other PCR inhibitors are all washed through the PP during the washing steps with ethyl alcohol. Finally, the DNA is eluted from the PP with deionized water.

### Analytical sensitivity

**Figure 3:**
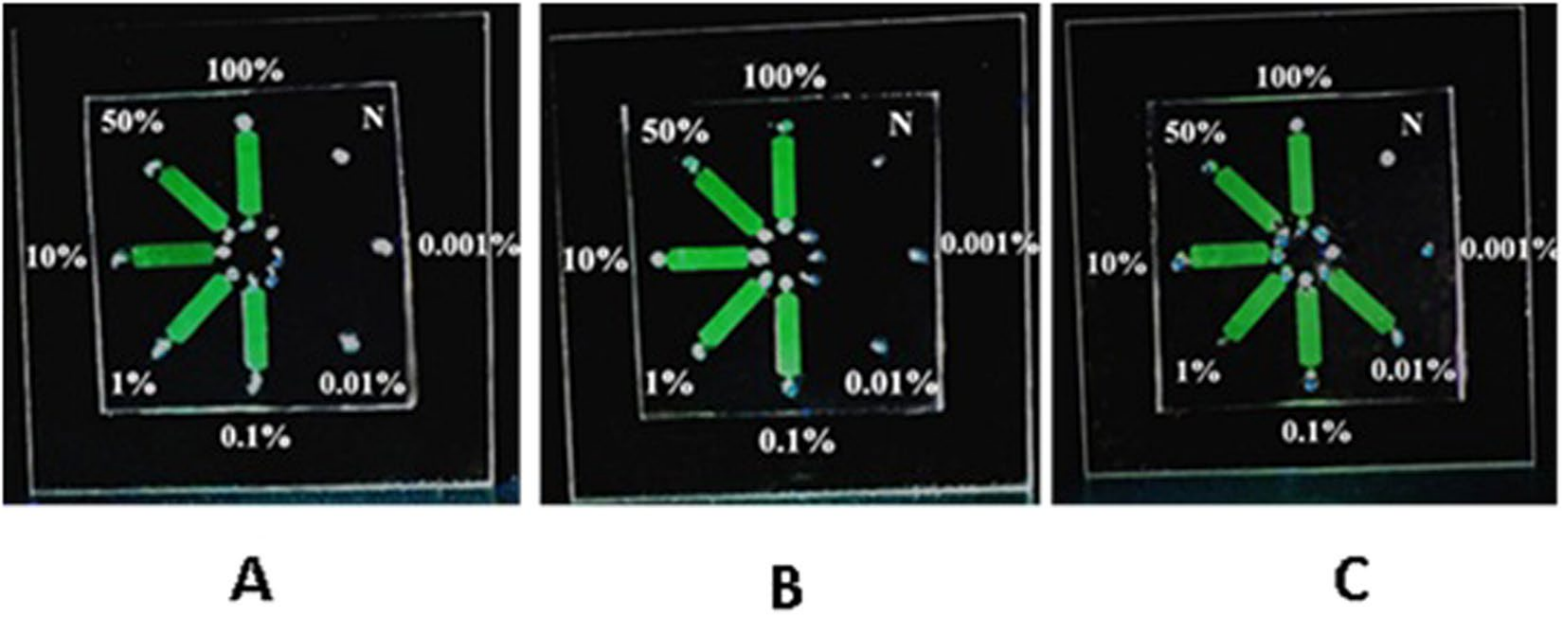
The detection limit for the JAK2 V617F and MPL W515K/L mutations in microchambers. Serial dilutions of mutant DNA at concentrations of 100%, 50%, 10%, 1%, 0.1%, 0.01%, 0.001%, and 0% (100% wild-type control) were analyzed by using the microchip-LAMP technique. A: JAK2 V617F; B: MPL W515K; C: MPL W515L.

To evaluate the ability of the LAMP microchamber to detect low amounts of JAK2 V617F, MPL W515K, and MPL W515L mutated DNA, we constructed a PDMS-glass hybrid microchip with eight 7 µL microchannels (Fig. 3). Operation of the microchip was simple did not require the use of any precise valves or pumps. We examined the detection limit using DNA that contained mixtures of wild-type and mutant JAK2 V617F, MPL W515K, and MPL W515L DNA in various ratios. We examined the detection limit using DNA that contained mixtures of wild-type and mutant JAK2 V617F, MPL W515K, and MPL W515L DNA in various ratios. Serial dilutions of mutant DNA at concentrations of 100%, 50%, 10%, 1%, 0.1%, 0.01%, 0.001%, and 0% (100% wild-type control) were analyzed by microchambers-based LAMP. To prepare the standards with 100%, 50%, 10%, 1%, 0.1%, 0.01%, 0.001% of mutations DNA load in a 20 ng/μL, 10 ng/μL and 2 ng/μL background concentration, respectively. Each mixture for accuracy of the dilution was analyzed three times using microchambers-based LAMP. The sensitivity of the microchip was visually evaluated by the naked eye. The sample containing 0.5 μL of nucleic acid was first introduced via the inlet. A reaction mixture for LAMP of 6.5 μL was drawn slowly into the microchannel by capillary force. The inlet and outlet were sealed with epoxy to form an integral microchamber for the LAMP reaction. The entire microfluidic chip was incubated at 63 °C for 1h, using a portable mini temperature control instrument. During the LAMP amplification process, visual confirmation can be achieved with the naked eye by adding a mixture of calcein that is quenched by manganese ions before the LAMP amplification^22^. The results from the 0.01–100% mutant samples were visible through use of a portable UV pen. These results of serial dilutions of mutant DNA in the 20 ng/μL, 10 ng/μL and 2 ng/μL background concentrations were concordant. As shown in Fig. 3, as little as 0.01% of the JAK2 V617F mutation, 0.1% of the MPL W515K mutation, and 0.1% of the MPL W515L could be detected, as a result of the merits of loop-mediated mechanism of the amplification. Furthermore, the calcein in the reaction mixture made the naked eye detection powerful and effective. We believe that this method has great potential for developing point-of-care devices.

### Accuracy of the LAMP assay

**Figure 4:**
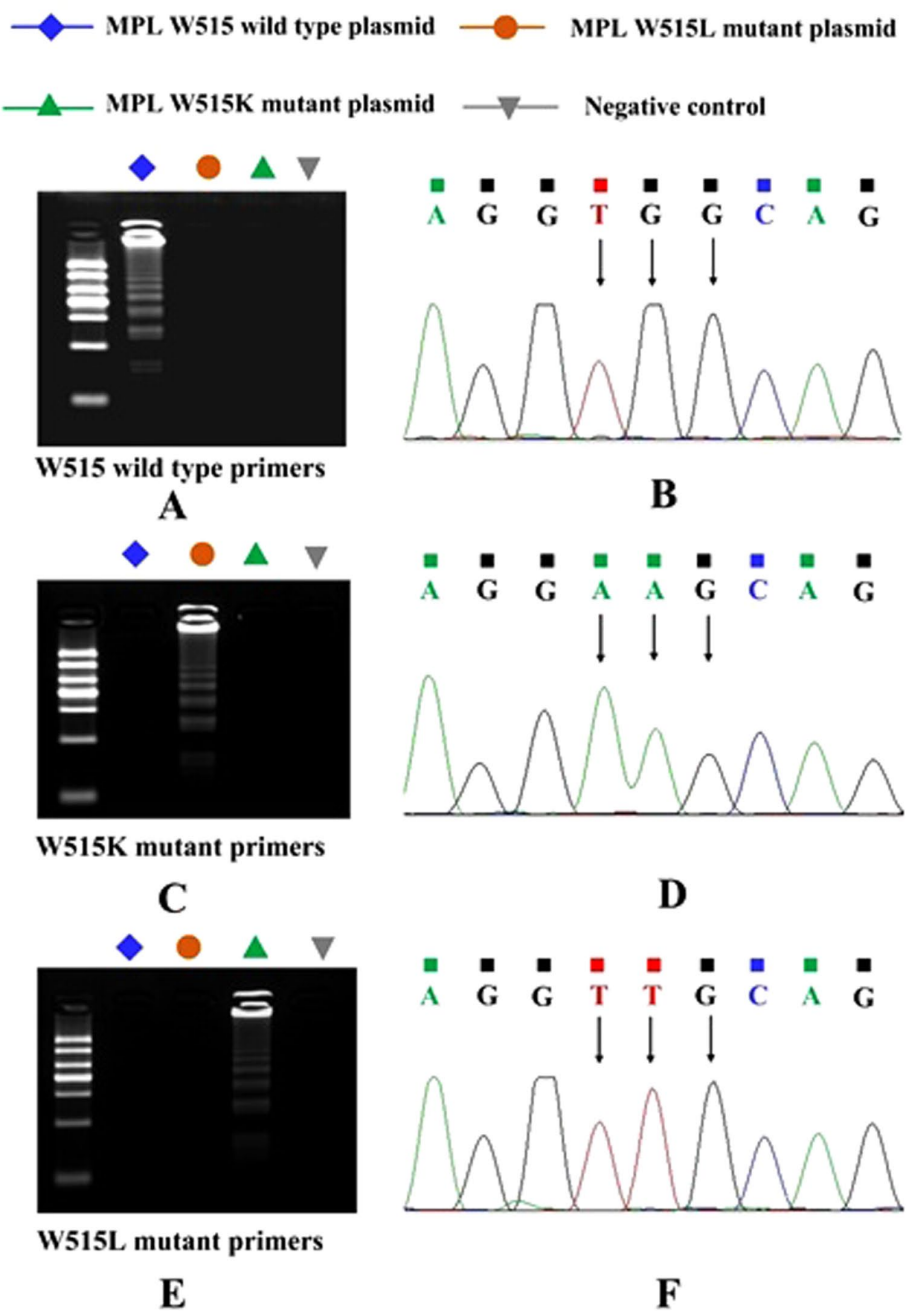
Accuracy of microchip-based LAMP for detection of the MPL W515K/L mutation. (**A**) Agarose gel electrophoresis of the microchip-based LAMP-amplified products with the MPL W515 wild-type primer. MPL W515 wild-type plasmid (blue), MPL W515K mutant plasmid (orange), MPL W515L mutant plasmid (green), and negative control (gray). (**B**) Sequencing results of the LAMP-amplified products from A. The arrowheads indicate the location of the point mutations. The wild-type sequence is TGG at this location (MPL W515 wild-type plasmid). (**C**) Agarose gel electrophoresis of the microchip-based LAMP-amplified products from the MPL W515K mutant primer. MPL W515 wild-type plasmid (blue), MPL W515K mutant plasmid (orange), MPL W515L mutant plasmid (green), and negative control (gray). (**D**) Sequencing results of the LAMP-amplified products from C. The arrowheads indicate the location of the point mutation. The mutated sequence is AAG at this location (MPL W515L plasmid). (**E**) Agarose gel electrophoresis of the microchip-based LAMP-amplified products with the MPL W515L mutant primer. MPL W515 wild-type plasmid (blue), MPL W515K mutant plasmid (orange), MPL W515L mutant plasmid (green), and negative control (gray). (**F**) Sequencing results of the LAMP-amplified products from E. The arrowheads indicate the location of the point mutation. The mutated sequence is TTG at this location (MPL W515L plasmid).

To demonstrate the accuracy of the LAMP method, we chose the MPL W515 wild-type and MPL W515K/L mutation plasmids as target genes and prepared MPL W515 wild-type and MPL W515K/L mutant primers that met the LAMP requirements. No amplification of MPL W515K/L mutation plasmids was observed when the MPL W515 wild-type primers were used. For detection of the MPL W515K mutation plasmids, no amplification was detectable when the wild-type primers or MPL W515L mutation primers were used. For detection of the MPL W515L mutation plasmids, no amplification was detectable when the wild-type primers or MPL W515K mutation primers were used. Gel electrophoresis of the LAMP products further confirmed that the primers designed to detect each target gene amplified the appropriate plasmid fragments (Fig. 4A,C and E). To confirm these mutations at the nucleotide level, the LAMP products from the MPL W515 wild-type plasmid and the MPL W515K/L mutation plasmid were sequenced (Sangon Biotech Co. Ltd. Shanghai, China). As shown in Fig. 4, the arrow heads indicate the location of the point mutations. The MPL W515 wild-type sequence is TGG at this location (Fig. 4B), whereas in the MPL W515K and MPL W515L mutation sequences, these bases are AAG (Fig. 4D) and TTG (Fig. 4F), respectively. The sequences of the cloned DNA agreed perfectly with the expected nucleotide sequences.

**Figure 5:**
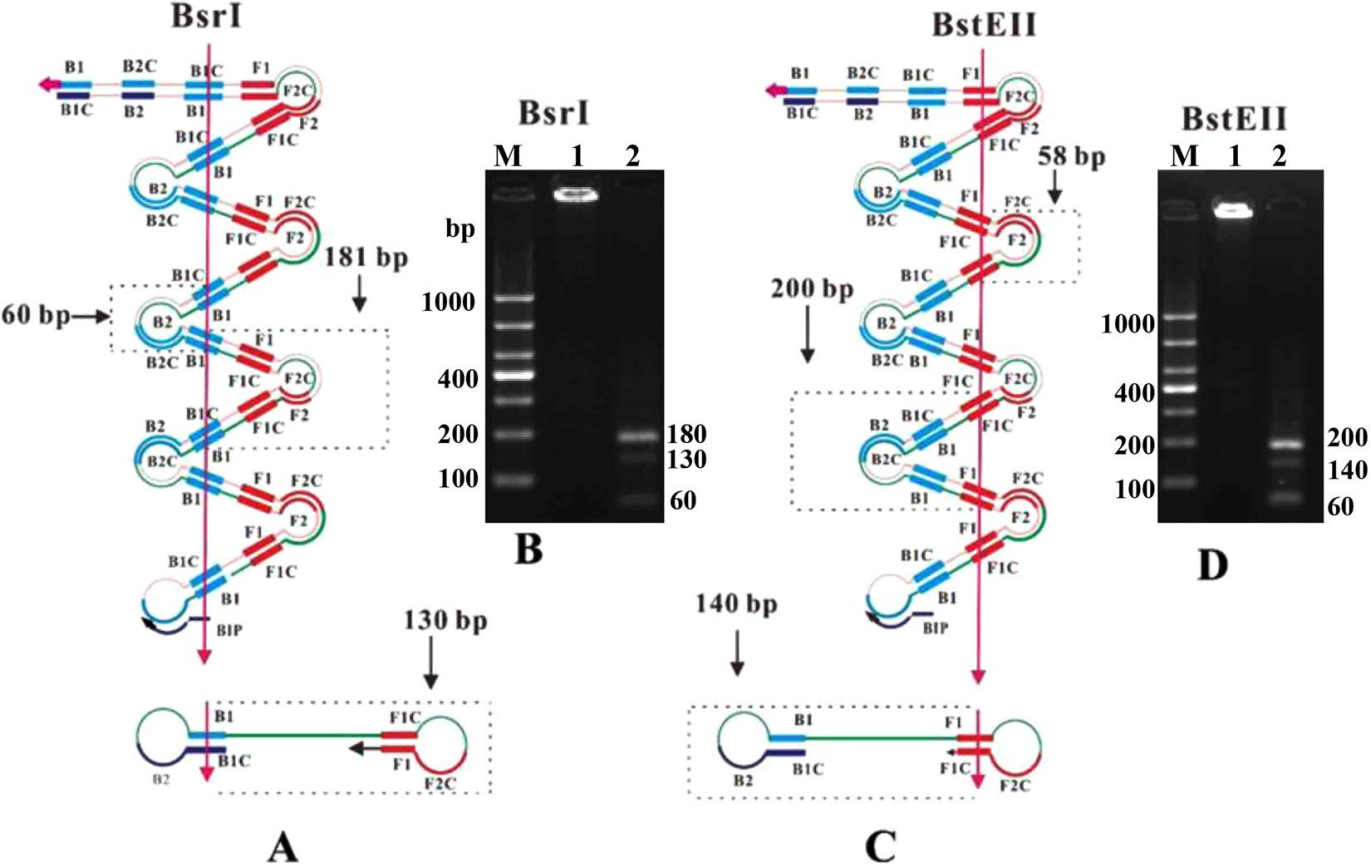
Restriction analysis of the amplified MPL W515K/L plasmid. The MPL W515K mutation plasmid amplified products were digested with Bsr I. (**A**) Bsr I cuts B1. Theoretically, the products would be fragmented to 60, 130 and 181 bp fragments by Bsr I. (**B**) The specific amplification confirmed by Bsr I. Lane M: 1000 bp ladder size markers; lane 1: ladder-like bands of the microchip-based LAMP products; lane 2: three bands of the predicted sizes of approximately 180 bp, 130 bp, and 60 bp produced by the Bsr I enzyme. The amplified products of the MPL W515L mutation plasmid were digested with BstEII. (**C**) BstEII cuts F1. Theoretically, the products should be fragmented to 58, 140 and 200 bp fragments by BstEII. (**D**) The specific amplification was confirmed by using BstEII. Lane M: 1000 bp ladder size markers; lane 1: ladder-like bands of the microchip-based LAMP products; lane 2: three bands of the predicted sizes of approximately 200 bp, 140 bp, and 60 bp products produced by BstEII.

To further confirm the accuracy of the method, the amplified products were digested with restriction endonucleases, and their sizes were analyzed by using gel electrophoresis. The final products were a mixture of stem-loop DNAs with various stem lengths and cauliflower-like structures with multiple loops formed by annealing between alternately inverted repeats of the target sequence in the same strand^23^. The products of the MPL W515K mutation plasmid amplification were digested with Bsr I. As shown in Fig. 5A, Bsr I cut B1. Consequently, if the amplification products were to have exactly the same structures, the products would be expected to be fragmented to 60, 130 and 181bp fragments by Bsr I. The sizes of the fragments generated by Bsr I digestion were approximately 60, 130, and 180 bp (Fig. 5B, lane 2), in good agreement with the predicted sizes. The amplified products of MPL W515L were digested by Bst E II. As shown in Fig. 5C, Bst-EII cuts F1. Theoretically, the products would be fragmented to 58, 140 and 200 bp fragments by Bst-EII. The sizes of the fragments generated by the Bst-EII digestion were approximately 60, 140, and 200 bp (Fig. 5D, lane 2), results in good agreement with the predicted sizes. We chose HEL and HL-60 as target DNA and prepared JAK2 V617F wild type and JAK2 V617F mutant primers that met the LAMP requirements. The results of accuracy analysis were consistent with what we have reported^14^. These results demonstrate that microchip-based LAMP is a highly accurate method for screening for the MPL W515K/L and JAK2 V617F mutations.

### Clinical sample analysis

Peripheral blood samples were collected from 40 patients with PV, 50 patients with ET, and 10 patients with PMF. To validate the performance of the method, the µmLchip assays were then evaluated by using mutation analysis of peripheral blood samples from 100 MPN patients who had previously been analyzed with unlabeled probe melting curve analysis (MCA) or real-time PCR. As shown in Table 1, among the 50 ET patients, 3 cases were identified to have MPL 515 mutations by using the µmLchip procedures, including 1 case with MPL W515K and 2 cases with MPL W515L. In addition, 22 cases were identified as JAK2 V617F mutation carriers by using the µmLchip procedures. Additionally 4 cases with JAK2 V617F and 1 case with MPL W515 K were identified in 10 PMF patients by using the µmLchip procedures. One ET patient was found to have concurrent MPL W515K and JAK2 V617F mutations. Among the 50 ET patients, 21 cases were identified as JAK2 V617F mutation carriers by unlabeled probe MCA. Among the 10 PMF patients, 4 cases with JAK2 V617F were detected by unlabeled probe MCA and 1 case with MPL W515 K was identified by using the real-time PCR. Thirty-seven out of 40 patients with PV (92.5%) were identified as JAK2 V617F mutation carriers by using the µmLchip procedures and unlabeled probe MCA, respectively. The results from the µmLchip were in perfect agreement with these results from the real-time PCR. To further demonstrate the accuracy of the µmLchip assay, the statistical analysis of the results of the µmLchip assay and the unlabeled probe MCA was performed using the Kappa analysis. Kappa value was 0.979 (Table SI2), which indicated the µmLchip assay was better consistent with the unlabeled probe MCA.

**Table 1:** Performance evaluation of the µmLchip assay.

Methods	ET (n = 50)				PMF (n = 10)				PV (n = 40)	
	515K+	515L+	617 F+	—	515 K+	515L+	617 F +	—	617 F+	—
MCA			21	26			4	5	37	3
Real-time PC**R**	1	2			1					
µmLchip	1	2	22	25	1		4	5	37	3

**Figure 6:**
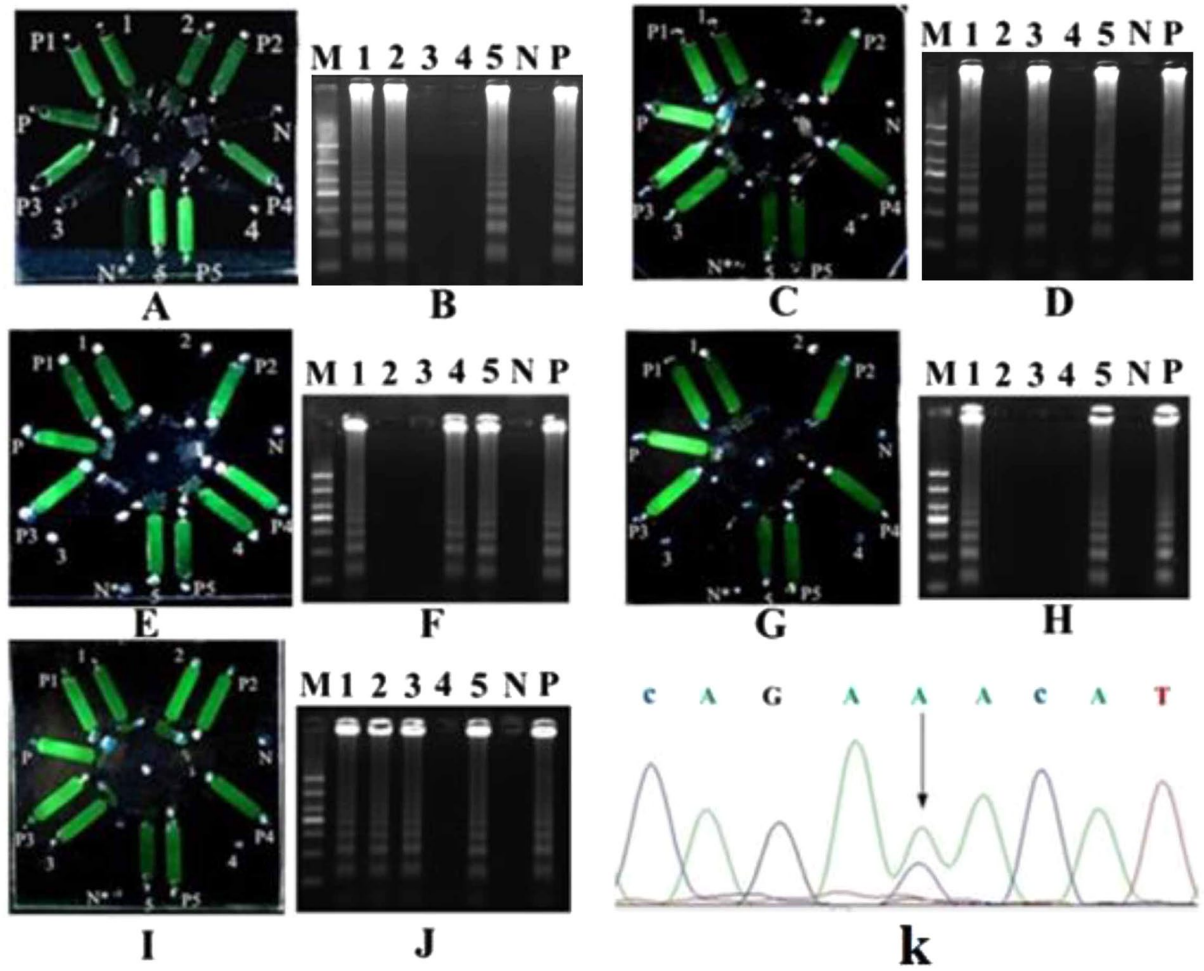
Performance of the µmLchip in the analysis of clinical samples. A patient with a heterozygous JAK2 V617F mutation (**A**) the result of the chip-based LAMP; (**B**) agarose gel electrophoresis of the LAMP-amplified products from Fig. 6A); a patient with the MPL W515K mutation (**C**) the result of the chip-based LAMP; D: agarose gel electrophoresis of the LAMP-amplified products from Fig. 6C); a patient with the MPL W515L mutation (**E**) the result of the chip-based LAMP; (**F**) agarose gel electrophoresis of the LAMP-amplified products from Fig. 6E); a patient with JAK2 V617F and MPL W515 wild-type (**G**) the result of the chip-based LAMP; (**H**) agarose gel electrophoresis of the LAMP-amplified products from Fig. 6G); and a patient with both MPL W515K and JAK2 V617F mutations (**I**) the result of the chip-based LAMP; (**J**) agarose gel electrophoresis of the LAMP-amplified products from Fig. 6I). The sequencing results of cloned PCR products are also shown (Fig. 6K). Lanes 1–5 represent the chambers: JAK2 V617F wild-type, JAK2 V617F mutation, MPL W515K mutation, MPL W515L mutation, and MPL W515 wild-type, respectively. Lane M, 1000 bp ladder size markers. Microchambers P1-P5 represent the positive control for microchamber 1–5, respectively. The microchamber N* represent the negative control.

There was one discrepant result, which was negative in the unlabeled probe MCA but positive in the µmLchip. After T-A cloning, the sequences of the cloned PCR products revealed the JAK2 V617F mutation in the sample (Fig. 6K). Some research reports suggested that LAMP for gene mutation detection is more sensitive and specific than conventional PCR methods^24, 25^. However, the small sample size make it difficult to draw concrete conclusions that the µmLchip assay displays a better diagnostic performance than unlabeled probe melting curve analysis in the diagnosis of gene mutations. Further studies to analyze a large number of samples are currently being performed by our group. Representative results of the assay are presented in Fig. 6. Figure 6A shows the results of a patient with a heterozygous mutation in the JAK2 V617F. As shown, the green fluorescence signal appears in chamber 1 (JAK2 V617F wild -type primers), chamber 2 (JAK2 V617F mutation primers), chamber 5 (MPL W515 wild-type primers), and chamber P (positive control) but not in chamber N (negative control), chamber 3 (MPL W515K mutation primers), or chamber 4 (MPL W515L mutation primers). The results were further confirmed by standard agarose gel electrophoresis (Fig. 6B). The results for a patient with the MPL W515K mutation (Fig. 6C,D), a patient with the MPL W515L mutation (Fig. 6E,F), a patient with the JAK2 V617F and wild-type MPL W515 (Fig. 6G,H), and a patient with concurrent MPL W515K and JAK2 V617F mutations (Fig 6I,J) are also shown.

The µmLchip assay established in our study used direct naked eye detection and was highly sensitive and accurate. Therefore, we believe that the portable microfluidic platform has great potential for the POCT of a wide range of diseases, especially in developing countries. It should be noted that CALR mutations have been described by some researchers in a majority of patients who had myeloproliferative neoplasms without mutations in JAK2 or MPL^26–28^. However, CALR mutation hasn’t been included in the diagnostic criteria for MPN until recently. The diagnosis and the management of patients with MPN have evolved since the identification of mutations that activate the JAK pathway (JAK2, CALR, and MPL mutations)^29^. Further studies on simultaneous analysis of these mutations on a microchip are going on in our group.

## Conclusions

We developed a portable microfluidic platform for rapid molecular diagnostic testing in patients with myeloproliferative neoplasms. The µmLchip combination of PP/capillary was functionalized for peripheral blood collection, rapid DNA release, amplification and direct naked-eye result read-out of the results. This method is highly sensitive and accurate. It should be noted that additional MPL mutant cases would need to be identified and run in order to fully demonstrate the robustness of the assay, but that the rates of detection in the assay are in line with other studies.The portable microfluidic platform may be very attractive in developing point-of-care diagnostics for MPL W515K/L and JAK2 V617F mutations. This method not only provides a diagnostic platform for MPN, but also provides a robust tool for MPN-induced stroke screening.

## Methods

### Microfluidic chip design and fabrication

**Figure 7:**
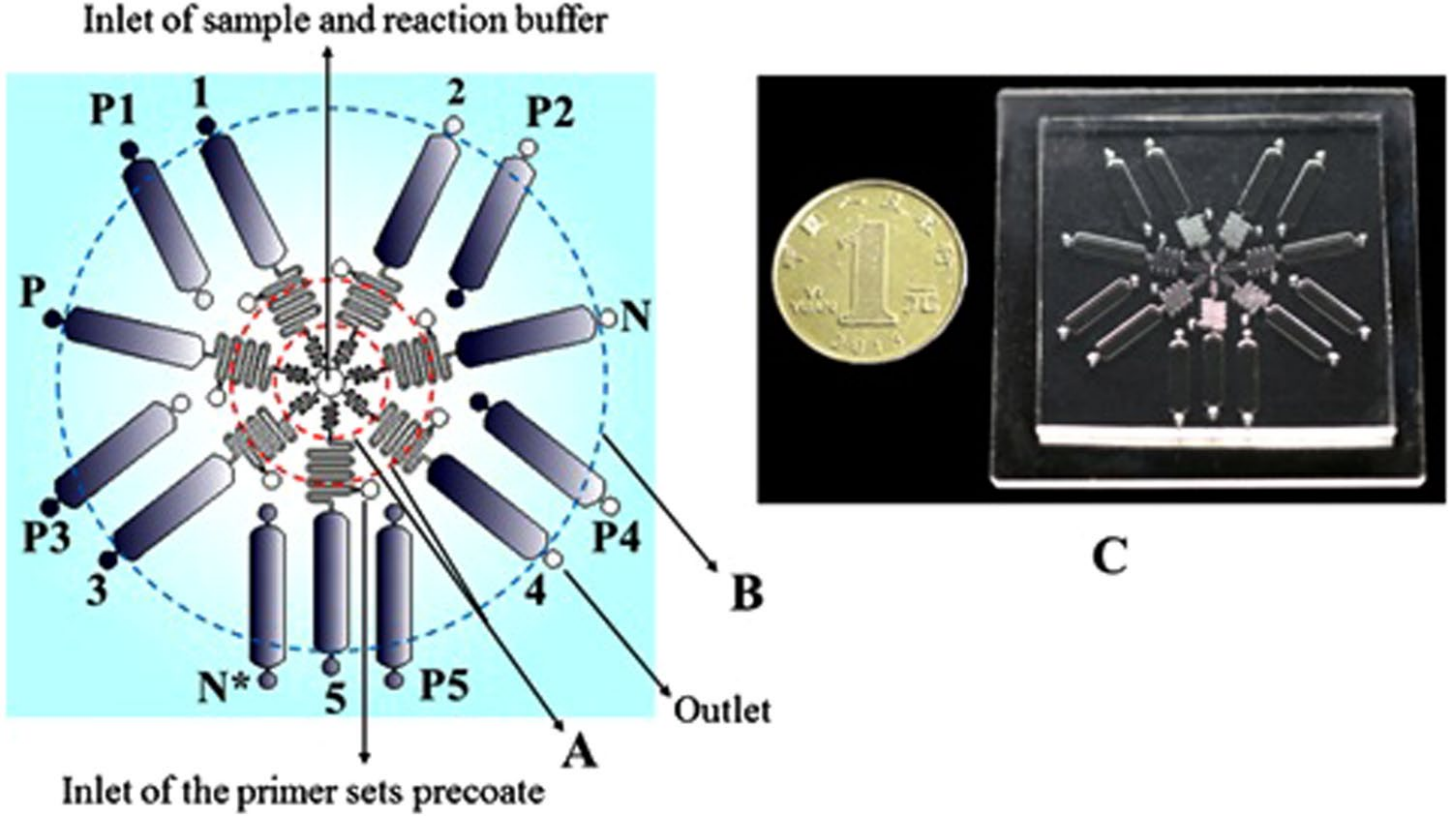
Structure diagram of the µmLchip and photograph of the chip system in a PDMS-glass format. (**A**) the region for controlling the flow of fluids; (**B**) the LAMP region; (**C**) a photograph of a µmLchip. Microchambers 1, 2, 3, 4, 5, P, and N (length, 10 mm; width, 2.4 mm; depth, 0.3 mm) were connected to the corresponding serpentine channels (length, 29.5 mm; width, 0.1-0.2 mm; depth, 0.05-0.3 mm) and were coated with wild-type JAK2 V617-primer sets, mutant JAK2 V617 primer sets, mutant MPL W515K primer sets, mutant MPL W515L primer sets, wild-type MPL W515 primer sets, human β actin primer sets, and no primer sets, respectively. The microchamber P loaded with human β actin-probes was used as a positive control, whereas the chamber N with no probes served as the negative control. Microchambers P1, P2, P3, P4 and P5 (length, 10 mm; width, 2.4 mm; depth, 0.3 mm) represent the positive controls for microchambers 1, 2, 3, 4, and 5, respectively. The microchamber N* represent the negative control.

As shown in Fig. 7, the µmLchip described here had two distinct functional domains, one for controlling the flow of fluid (region (A), and the other for LAMP reaction (region (B). The region that controlled the flow of the fluids contained a sample inlet and 7 serpentine channels (length, 29.5 mm; width, 0.1–0.2 mm; depth, 0.05-0.3 mm). The LAMP domain contained six independent microchambers (P1, P2, P3, P4, P5, and N*) and seven microchambers (length, 10 mm; width, 2.4 mm; depth, 0.3 mm) that were connected to the corresponding serpentine channels (length, 29.5 mm; width, 0.1–0.2 mm; depth, 0.05-0.3 mm). Some microchambers were coated with certain quantities of specific LAMP primer sets by using the flow patterning technique. Microchamber 1, 2, 3, 4, and 5 were coated with wild type JAK2 V617F primer sets, mutant JAK2 V617 primer sets, mutant MPL W515K primer sets, mutant MPL W515L primer sets, and wild type MPL W515 primer sets, respectively. These functionalized microchannels recognized the specific nucleic acid fragments of the JAK2 V617F and MPL W515K/L gene mutation *in situ*, thus permitting rapid amplification and simultaneously producing the LAMP signal. We also functionalized microchamber P with human β actin primer sets as a positive controls, whereas microchamber N contained no primers as the negative control. Microchamber P1, P2, P3, P4 and P5 (length, 10 mm; width, 2.4 mm; depth, 0.3 mm) represent the positive controls for microchamber 1, 2, 3, 4, and 5, respectively. The microchamber N* represent the negative control. The microchamber P loaded with human β actin-probes was used as a positive control, whereas chamber N, which contained no probes, served as the negative control. It should be noted that a SU-8 layer with a thickness of approximately 150 µm could be achieved by spin-coating SU-8 3050 at 1000 rpm for 30 s. A thickness of 50 µm could be achieved by spin-coating SU-8 3050 at 4000 rpm for 30 s (MA6, Karl Suss Corp., GER).

The PDMS/glass hybrid microfluidic chip was produced by molding a PDMS silicone elastomer against a microfabricated master^30, 31^. The PDMS precursor mixture was prepared at a weight ratio of base to curing agent of 10:1, and was poured carefully on the master, placed under vacuum for 0.5 h to remove the bubbles, and cured at 85 °C for 2 h. The cured PDMS replica was gently peeled off the master and irreversibly sealed to a glass slide, using plasma, to form a leak-proof integral chip. The entire chip was then heated at 120 °C for 30 min to remove any contaminants.

### Developing a handheld battery-powered heating device

**Figure 8:**
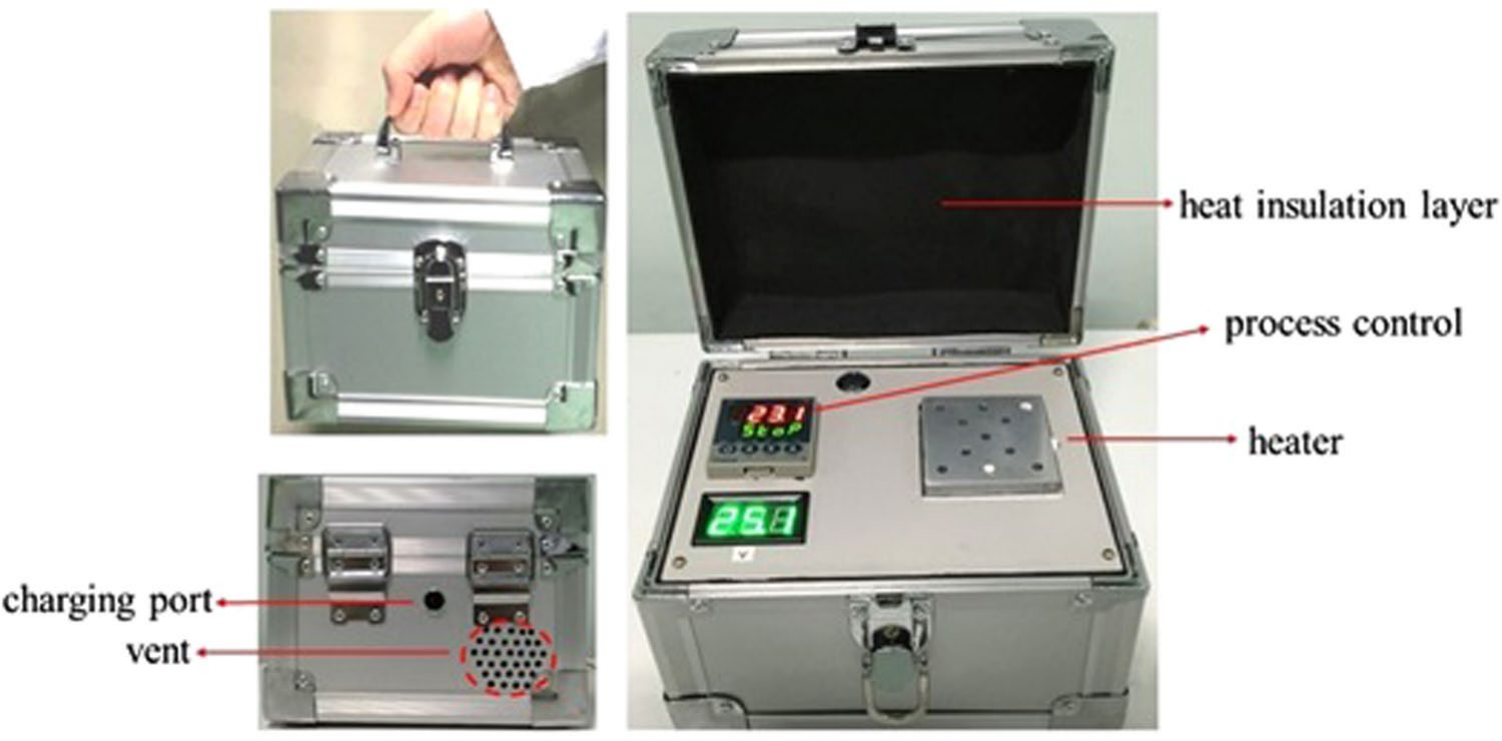
A photograph of the handheld battery-powered heating device. The main components of the handheld battery-powered heating device are a process-controlling module and a metal heater module. There are vents and charging port on the back of the device and a heat insulation layer on the top of the device.

The main components of the handheld battery-powered heating device (length, 15 cm; width, 12 cm; height, 10 cm) consisted of two modules, a process-controlling module and a heater module (Fig. 8). The metal heater (length, 6 cm; width, 6 cm) had 9-holes (diameter, 4 mm; depth, 6 cm), which were required for the PP/capillary extraction of the nucleic acid. There are vents and charging port on the back of the device and a heat insulation layer on the top of the device. The main technical parameters for the device were: temperature range, 37 °C–100 °C; temperature fluctuation (°C), ±0.3.

### Polypropylene matrix (PP)/capillary fabrication and operation

As shown in Fig. 4A, the glass capillary described here has three distinct functional domains, one for sampling (length, 4 cm; external diameter, 0.7 mm; internal diameter 0.5 mm), another for DNA extraction (the region filled with of 8 mg PP, diameter, 6 mm; external diameter, 0.7 mm; internal diameter 0.5 mm) and the last for controlling the flow of fluids (length, 2 cm; external diameter, 1.2 mm; internal diameter 1.0 mm).A hand-held rubber dropper can serve as a general purpose small-volume liquid -handling platform for DNA extraction. Cell lysates was prepared by using 10 M Tris-Cl (pH 8.0), 15 mM EDTA (pH 8.0), 0.4% SDS, and 200 µg/mLProteinase K. The DNA extraction efficiency of the PP/capillary was tested using whole blood. The extraction procedure consisted of sampling, cell lysis, and elution steps. First, the whole blood sample was siphoned onto the PP, and then 100 µl cell lysates were loaded into the region of DNA extraction by using the hand-held rubber dropper. After sealing of both ends of the PP/capillary with rubber plugs, the PP/capillary was incubated at 56 °C for 5 min in a hole of the handheld heating device to complete the cell lysis. The rubber plugs were then removed from the PP/capillary. A 250 µL volume of the wash solution (ethyl alcohol) was used to wash away the proteins or other contaminants. Finally, the DNA was eluted from the PP with 50 µL deionized water, and 4 µL aliquots were injected into the µmLchip for subsequent LAMP analysis with a pipettor. The overall extraction process can be completed in only 6 minutes, including 5 min of incubation for cell lysis and 1 min of washing.

### Samples collection

This study was approved by the ethics committees of Fudan University Huashan Hospital and samples were collected only after the participants had given their informed consent. Peripheral blood samples were collected from 40 patients with PV, 50 patients with ET, and 10 patients with PMF and all experiments were performed in accordance with relevant guidelines and regulations.

### LAMP amplification

The LAMP reaction in this work was carried out using the Loopamp® reaction mixture. The entire volume of the system was 50 μL, which contained 20 mMTris-HCl (pH 8.8), 10 mMKCl, 8 mM MgSO4, 10 mM (NH4)_2_SO4, 0.1% Tween 20, 0.8 M betaine, 25 mM calcein, 0.5 mM MnCl_2_, 1.4 mMdNTPs, 0.2 mM each of the outer primers (F3/B3), 1.6 mM each of the inner primers (FIP/BIP), 1.6 mM each of the loop primers (LF), 8 U of Bst Polymerase, 2 μL of calcein (25 mM), and 4 μL of DNA as a template. The amplification was performed at 63 °C in a water bath for 60 min. The primers were designed manually according to the primer design software Primer Explorer V5. The details of the LAMP primers used in this study are listed in Table SI1.

### On-chip LAMP procedures

The assay was first optimized in a tube, and then translated to the chip format. After the LAMP mix was prepared in a biosafety cabinet, both 50 µL of the LAMP mix and 6 µL of mineral oil were subsequently introduced at the central hole to fill the various different LAMP zones. After the inlet and outlet reservoir were sealed with Epoxy, the microfluidic device was placed on the handheld battery-powered heating device at 63 for 60 min for the LAMP reactions. After the LAMP reactions, a portable UV pen light was applied to illuminate the LAMP products. The generated fluorescence was captured by using a cellular phone camera (e.g., iPhone 5).

### Unlabeled probe melting curve analysis for JAK2 V617F mutation

The unlabeled probe melting curve analysis for the JAK2 V617F mutation was performed as we have described previously^32^. The analysis method for the genotyping of JAK2 V617F was performed by using a Rotor-Gene® Q real-time PCR system. To prevent the extension of the probe during PCR, a 3′- carbon based C3 blockage was introduced. Unlabeled probe melting analysis was developed on the basis of asymmetric PCR. After asymmetric PCR, a large number of superfluous single strands hybridize with the unlabeled probe. The part of the curve in the low melting temperature represents the region of probe and product. High resolution melting (HRM)-curve analysis was performed with Rotor-Gene® Q 1.7 software. Each assay contained positive and negative controls (homozygous wild type, heterozygous, and homozygous mutant).

### Real-Time PCR Assay for MPLW515 K/L mutations

Real-time PCR was performed using a Mastercycler PCR system (Eppendorf, Germany). Three different real-time reactions were set up in triplicate (one each for W515L, W515K, and wild-type control) for each DNA sample. The probes were designed according to procedures described by Pancrazzi and colleagues^33^. The sequences of the forward and reverse primers for PCR were 5′-TAGCCTGGATCTCCTTGGTG-3′ and 5′-ACAGAGCGAACCAAGAATGC-3′, respectively. The 20 µl reactions contained 10 µl Premix HotStart (Takara, Japan), 300 nmol/L of each primer, 150 nmol/L each LNA-modified probe, and 40–60 ng DNA. Control wells without template (NTC) were included in each assay. Amplification and detection were performed under the following conditions: hold at 95 °C for 10 minutes followed by 40 cycles at 94 °C for 30 seconds and 66 °C for 30 seconds.

## Electronic supplementary material

Supplementary Information
