# Supplementary material for: A portable microfluidic platform for rapid molecular diagnostic testing of patients with myeloproliferative neoplasms
Source: Sci Rep. 2017 Aug 17;7:8596. doi: 10.1038/s41598-017-08674-8 (PMC5561050; doi:10.1038/s41598-017-08674-8)
Supplement: Supplementary file 1 — Supplementary Information [file 41598_2017_8674_MOESM1_ESM.pdf]

A portable microfluidic platform for rapid molecular diagnostic testing of patients with myeloproliferative neoplasms

Hua Wang[1], Xinju zhang[1], Xiao Xu[1], Qunfeng Zhang[1], Hengliang Wang[2], Dong Li[3], Zhihua Kang[1], Zhiyuan Wu[1], Yigui Tang[1], Zhenhua An[2], and Ming Guan[1*]

1 Dept. of Laboratory Medicine, Huashan Hospital, Shanghai Medical College, Fudan University, Shanghai, 200040, China

2 Dept. of Physics, Fudan University, Shanghai, 200040, China

3 Dept. of Clinical Laboratory, Shanghai Tongji Hospital, Tongji University School of Medicine, Shanghai, 200065, China

Corresponding author

Prof. Ming Guan, E-mail: guanming88@yahoo.com, Fax: +86 21-6248-1061; Tel: +86 21-5288-8048

Support information

1. Primer sequences

Table SI1 Details of LAMP primers

Primer name	Sequence (5' to 3')
F3-K	TCCTAGCCTGGATCTCCTTG
B3-K	CGGTGGACGGAGATCTGG
FIP-K	TCCTCAGCAGCAGCAGGCCTGACCGCTCTGCATCTAGTG
BIP-K	AGGCAGGAGACTGGCGGTGTCACAGAGCGAACCAAGAA
LB-K	GACCAGGTGGAGCCGAA
F3-L	GGGGCTGGCTGGATGA
B3-L	GCGAACCAAGAATGCCTGTT
FIP-L	GCAGAGCGGTCACCAAGGAGATGGGTGGGCCGAAGTCT
BIP-L	TTGAAGTTTCCTGCACACTTGGTCCACCGCCAGTCT
LF-L	AGGCTAGGAGACAAAAAGGGTC
F3-W	GGGGCTGGCTGGATGA
B3-W	GCGAACCAAGAATGCCTGTT
FIP-W	GCAGAGCGGTCACCAAGGAGATGGGTGGGCCGAAGTCT
BIP-W	AGGCAGTTTCCTGCACACTTGGTCCACCGCCAGTCT
LF-W	AGGCTAGGAGACAAAAAGGGTC

Table SI2 Comparison of the JAK2 V617F mutation results using MCA and μmLchip (n = 100)

μmLchip	MCA		Subtotal
	617F +	617F-	
617F+	62	1	63
617F-	0	37	37
Subtotal	62	38	100

Kappa value was 0.979

2. Accuracy assay

In order to demonstrate the accuracy of the method, we chose MPL W515 wild type plasmid, MPL W515K mutation plasmid and MPL W515L mutation plasmid as target sequence and prepared W515 wild type and W515K/L mutant primers that met the LAMP requirements. LAMP reaction in this work was carried out using the Loopamp® reaction mixture. The whole volume of the system was 25 μl, which contained 20 mM Tris-HCl (pH 8.8), 10 mM KCl, 8 mM MgSO4, 10 mM (NH4)2SO4, 0.1% Tween20, 0.8 M Betaine, 25 mM Calcein, 0.5 mM MnCl2, 1.4 mM dNTPs, 0.2 mM each of the outer primer (F3/B3), 1.6 mM each of the inner primer (FIP/BIP), 1.6 mM each of the loop primer (LF-K/L/W), 8U Bst Polymerase, and with 2 μl of DNA as a template. The assay was performed at a constant temperature of 63 ℃ for 1 h on an LA-500 turbid meter (Teramecs Co, Kyoto, Japan) for real-time monitoring. Using the W515K mutation primer set (P-K), the W515K mutation sequences were amplified, while neither the W515L mutation nor the W515 wild type sequences were amplified (Fig. SI1). Using the W515L mutation primer set (P-L), the W515L mutation sequences were amplified, while neither the W515K mutation nor the W515 wild type sequences were amplified (Fig. SI2).Using the W515 wild type primer set (P-W), the W515 wild type sequences were amplified, while neither the W515K nor the W515Lmutation sequences were amplified (Fig. SI3). The results demonstrated the accuracy of the method.

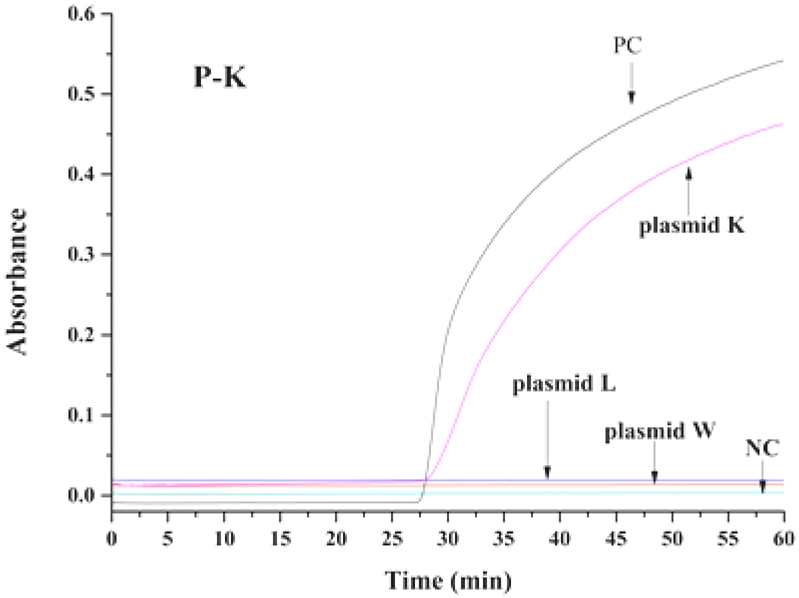

Fig.SI1 Amplification of the target sequence in W515 wild type and W515K/L mutation with the W515K mutation primer set monitored by a real-time turbid meter (turbidity at 650 nm). P-K: W515K mutation primer set; plasmid K: W515K mutation plasmid; plasmid L: W515L mutation plasmid; plasmid W: W515 wild type plasmid; NC: the negative control; PC: the positive control.

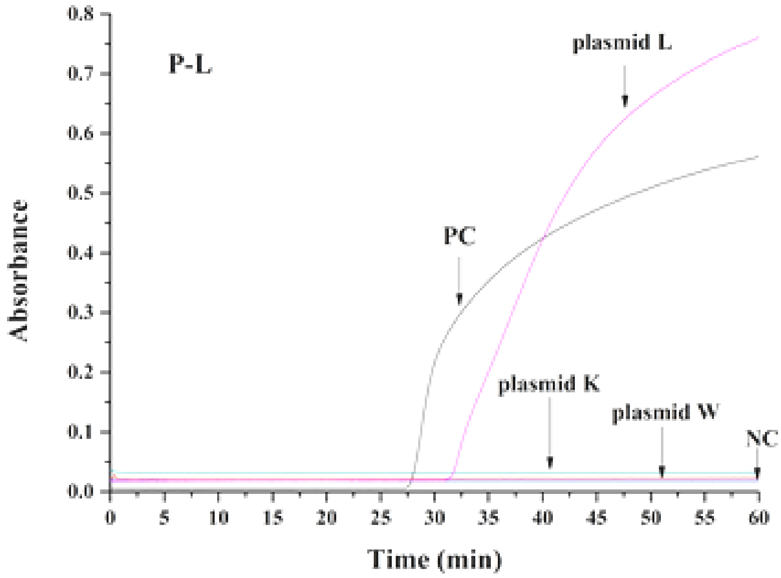

Fig.SI2 Amplification of the target sequence in W515 wild type and W515K/L mutation with the W515L mutation primer set monitored by a real-time turbid meter (turbidity at 650 nm). P-K: W515K mutation primer set; plasmid K: W515K mutation plasmid; plasmid L: W515L mutation plasmid; plasmid W: W515 wild type plasmid; NC: the negative control; PC: the positive control.

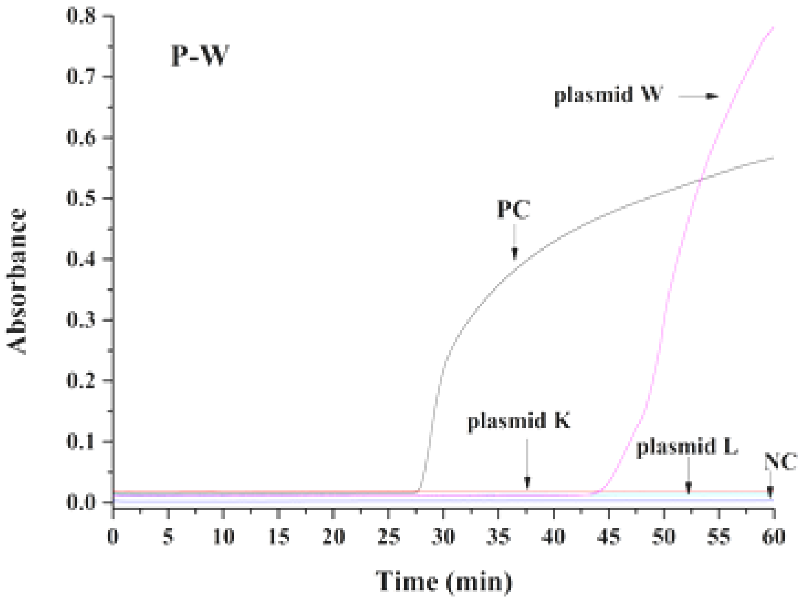

Fig.SI3 Amplification of the target sequence in W515 wild type and W515K/L mutation with the W515 wild type primer set monitored by a real-time turbid meter (turbidity at 650 nm). P-K: W515K mutation primer set; plasmid K: W515K mutation plasmid; plasmid L: W515L mutation plasmid; plasmid W: W515 wild type plasmid; NC: the negative control; PC: the positive control.

3. On-PP/capillary whole blood genomic DNA extraction

To provide a more comprehensive validation of the reliability of the DNA extraction system, the more replicates were tested from 10 patients with anemia and 10 healthy controls peripheral blood, at these volumes. Each sample was tested three times. As shown in Fig. S4, the result shows that the DNA concentrations of patients with anemia were significantly lower than that of healthy control.

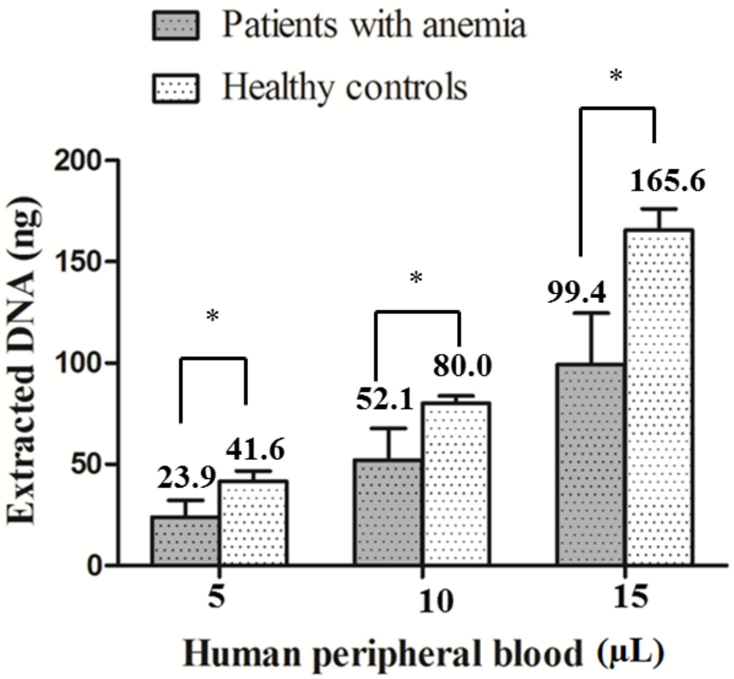

Fig.SI4. On-PP/capillary peripheral blood genomic DNA extraction.

Comparison of DNA extraction yields the group of patients with anemia and the group of healthy controls. With 5, 10, and 20 μL of peripheral blood, the group of healthy controls provides 41.6, 80.0, and 165.6 ng DNA, higher than those obtained by the group of patients with anemia (23.9, 52.1, and 99.4 ng). * $P < 0.01$.

Full-length gels and blots

1. The results of electrophoresis in an agarose gel (Figure 2C)

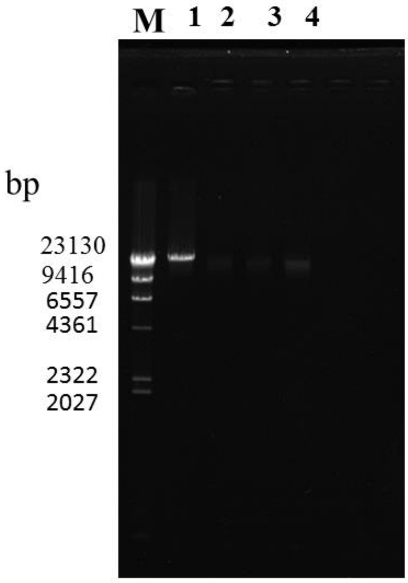

Figure 2C. The results of electrophoresis in an agarose gel. Lanes 2-4: the extraction of the genomic DNA fractions from 5, 10, and 20 μL of whole blood, respectively. Lane M: DNA marker, λ DNA/HindIII; Lane 1: λ DNA (50.0 kb).

2. Electrophoresis of PCR products amplified from 5, 10, and 20 μL of human peripheral blood extracted on the PP/capillary. (Fig 2D)

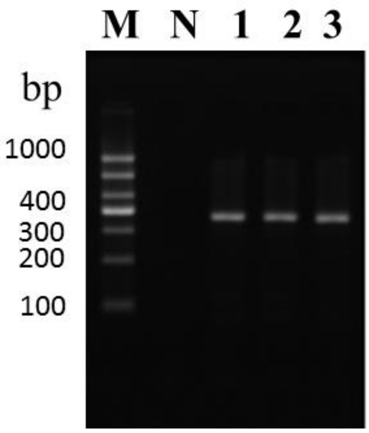

Figure 2D. Electrophoresis of PCR products amplified from 5, 10, and 20 μL of human peripheral blood extracted on the PP/capillary. Lane M: 1000 bp DNA marker; NC: negative control; Lanes 1-3: PCR-amplified results from 5, 10, and 20 μL of peripheral blood, respectively.

3. Electrophoresis of LAMP products amplified from 5, 10, and 20 μL of human peripheral blood extracted with the PP/capillary (Figure 2E).

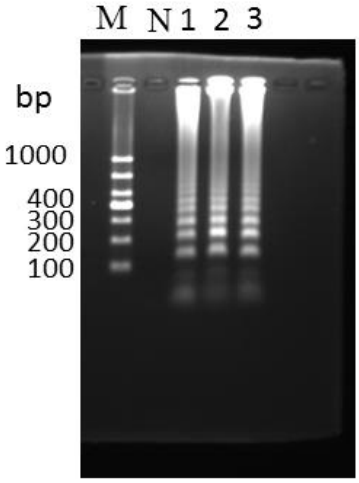

Figure 2E. Electrophoresis of LAMP products amplified from 5, 10, and 20 μL of

human peripheral blood extracted with the PP/capillary. Lane M: 1000 bp DNA marker; NC: negative control; Lanes 1-3: LAMP-amplified results from 5, 10, and 20 μL of peripheral blood, respectively.

4. Agarose gel electrophoresis of the microchip-based LAMP-amplified products with the MPL W515 wild-type primer (Figure 4A).

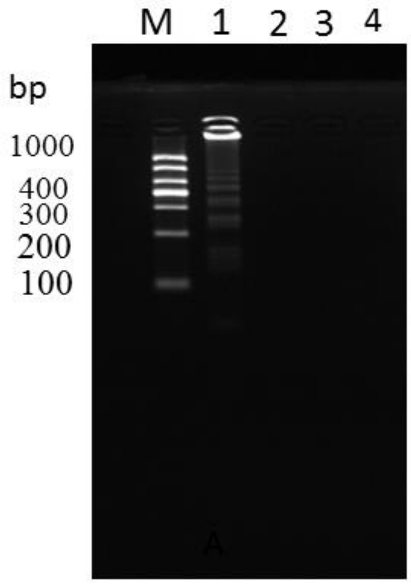

Figure 4A Agarose gel electrophoresis of the microchip-based LAMP-amplified products with the MPL W515 wild-type primer. MPL W515 wild-type plasmid (1), MPL W515K mutant plasmid (2), MPL W515L mutant plasmid (3), and negative control (4).

5. Agarose gel electrophoresis of the microchip-based LAMP-amplified products from the MPL W515K mutant primer (Figure 4C).

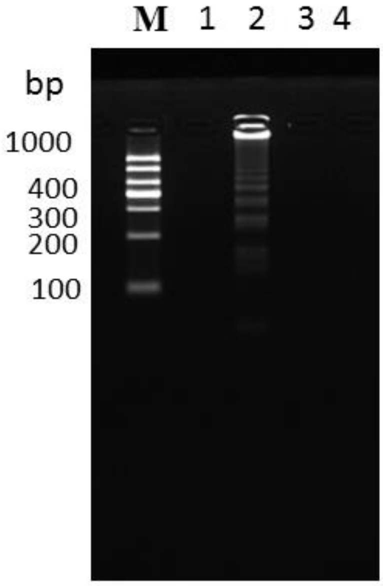

Figure 4C Agarose gel electrophoresis of the microchip-based LAMP-amplified products from the MPL W515K mutant primer. MPL W515 wild-type plasmid (1), MPL W515K mutant plasmid (2), MPL W515L mutant plasmid (3), and negative control (4).

6. Agarose gel electrophoresis of the microchip-based LAMP-amplified products with the MPL W515L mutant primer.

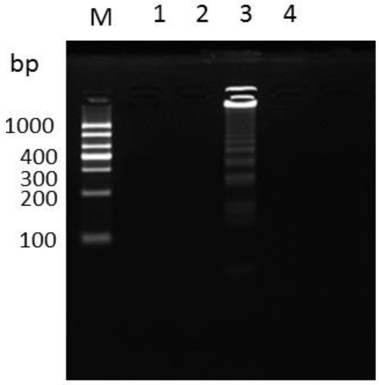

Figure 4E Agarose gel electrophoresis of the microchip-based LAMP-amplified products with the MPL W515L mutant primer. MPL W515 wild-type plasmid (1), MPL W515K mutant plasmid (2), MPL W515L mutant plasmid (3), and negative control (4).

7. The MPL W515K mutation plasmid amplified products were digested with Bsr I. (Figur 5B).

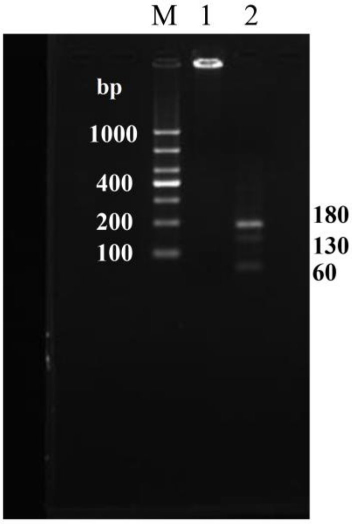

Figure 5B.The MPL W515K mutation plasmid amplified products were digested with Bsr I. The specific amplification confirmed by Bsr I. Lane M: 1000 bp ladder size markers; lane 1: ladder-like bands of the microchip-based LAMP products; lane 2: three bands of the predicted sizes of approximately 180 bp, 130 bp, and 60 bp produced by the Bsr I enzyme.

8. The amplified products of the MPL W515L mutation plasmid were digested with BstEII (Figure 5D)

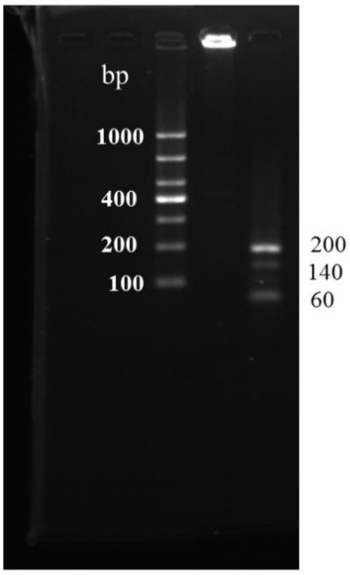

Figure 5D. The specific amplification was confirmed by using BstEII. Lane M: 1000 bp ladder size markers; lane 1: ladder-like bands of the microchip-based LAMP products; lane 2: three bands of the predicted sizes of approximately 200 bp, 140 bp, and 60 bp products produced by BstEII.

9. Heterozygous JAK2 V617F mutation (Figure 6B)

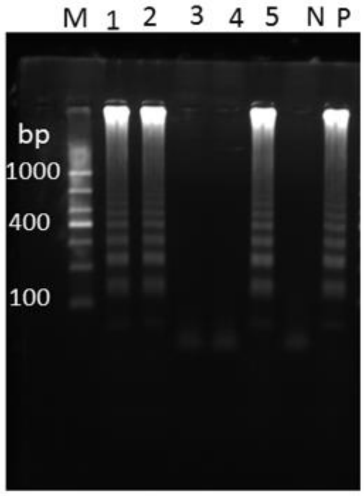

Figure 6B

10. MPL W515K mutation (Figure 6D)

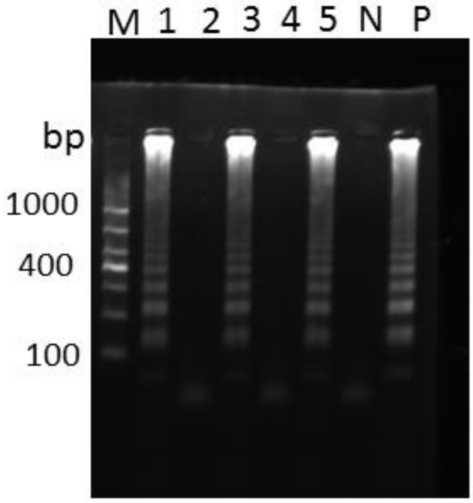

Figure 6D

11. MPL W515L mutatin (Figure 6F)

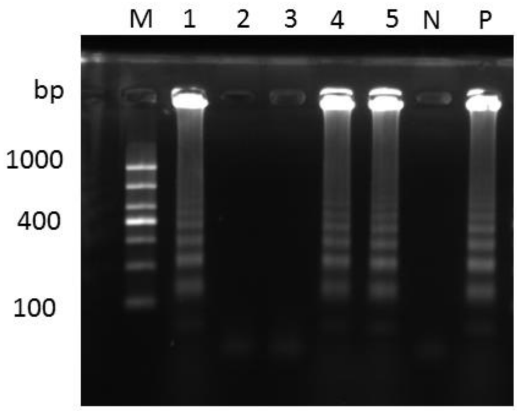

Figure 6F

12. JAK2 V617F and MPL W515 wild-type (Figure 6H)

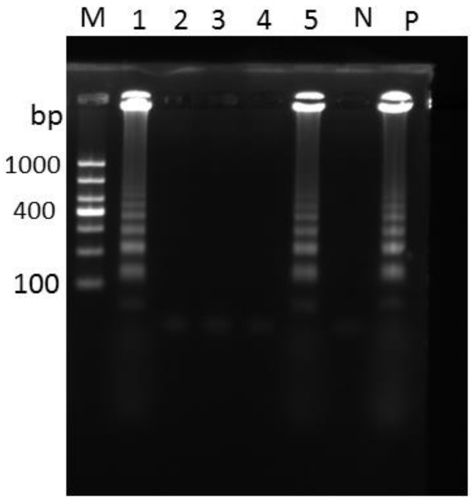

Figure 6H

13. MPL W515K and JAK2 V617F mutations (Figure 6J)

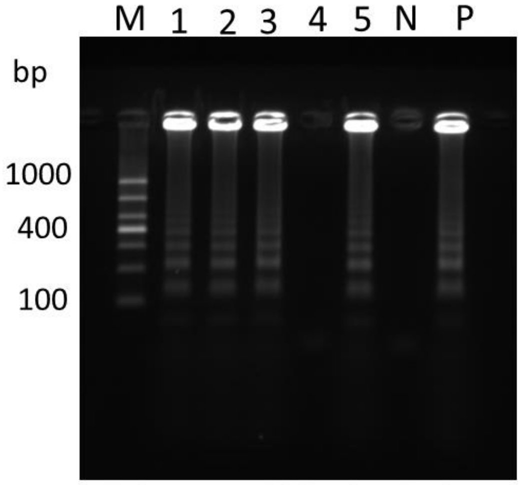

Figure 6J
